# Supplementary material for: Environmental health management in local community isolation facilities during COVID-19 pandemic: A case study in Nakhon Si Thammarat, Thailand
Source: PLoS One. 2025 Oct 6;20(10):e0333638. doi: 10.1371/journal.pone.0333638 (PMC12500105; doi:10.1371/journal.pone.0333638)
Supplement: S1 — (PDF) [file pone.0333638.s001.pdf]

COVID-19 Confirm case in Nakhon si thammarat 2020 - 2024

Year / Week	2021		
	Period	Case number	Accumulate case
1	3-Jan - 9-Jan 21		
2	10-Jan - 16-Jan 21		
3	17-Jan - 23-Jan 21		
4	24-Jan - 30-Jan 21		
5	31-Jan - 6-Feb 21		
6	7-Feb - 13-Feb 21		
7	14-Feb - 20-Feb 21		
8	21-Feb - 27-Feb 21		
9	28-Feb - 6-Mar 21		
10	7-Mar - 13-Mar 21		
11	14-Mar - 20-Mar 21		
12	21-Mar - 27-Mar 21		
13	28-Mar - 3-Apr 21		
14	4-Apr - 10-Apr 21		
15	11-Apr - 17-Apr 21	28	34
16	18-Apr - 24-Apr 21	105	139
17	25-Apr - 1-May 21		
18	2-May - 8-May 21		
19	9-May - 15-May 21	155	706
20	16-May - 22-May 21	91	797
21	23-May - 29-May 21	56	853
22	30-May - 5-Jun 21		
23	6-Jun - 12-Jun 21		
24	13-Jun - 19-Jun 21	335	1298
25	20-Jun - 26-Jun 21	138	1436
26	27-Jun - 3-Jul 21		
27	4-Jul - 10-Jul 21		
28	11-Jul - 17-Jul 21	274	1989
29	18-Jul - 24-Jul 21	624	2613
30	25-Jul - 31-Jul 21	910	3523
31	1-Aug - 7-Aug 21		
32	8-Aug - 14-Aug 21	832	5421
33	15-Aug - 21-Aug 21	766	6187
34	22-Aug - 28-Aug 21	1151	7338
35	29-Aug - 4-Sep 21		
36	5-Sep - 11-Sep 21		
37	12-Sep - 18-Sep 21	1976	11743
38	19-Sep - 25-Sep 21	2168	13906
39	26-Sep - 2-Oct 21		
40	3-0ct - 9-Oct 21		
41	10-Oct - 16-Oct 21	2815	21849
42	17-Oct - 23-Oct 21	3958	25807
43	24-Oct - 30-Oct 21	3854	29661
44	31-Oct - 6-Nov 21		
45	7-Nov - 13-Nov 21	2411	35064
46	14-Nov - 20-Nov 21	2559	37623
47	21-Nov - 27-Nov 21	2773	40396
48	28-Nov - 4-Dec 21		
49	5-Dec - 11-Dec 21		
50	12-Dec - 18-Dec 21	1607	46791
51	19-Dec - 25-Dec 21	1110	47901
52	26-Dec - 1-Jan 22	898	48799
53	2-Jan - 8-Jan 22		

Week \ Year	2022		
	Period	Case number	Accumulate case
1	2-Jan - 8-Jan 22		
2	9-Jan - 15-Jan 22	1315	51084
3	16-Jan - 22-Jan 22	1072	52156
4	23-Jan - 29-Jan 22	914	53070
5	30-Jan - 5-Feb 22		
6	6-Feb - 12-Feb 22		
7	13-Feb - 19-Feb 22	3682	59689
8	20-Feb - 26-Feb 22	5938	65627
9	27-Feb - 5-Mar 22		
10	6-Mar - 12-Mar 22		
11	13-Mar - 19-Mar 22	10422	89974
12	20-Mar - 26-Mar 22	10335	100309
13	27-Mar - 2-Apr 22		
14	3-Apr - 9-Apr 22		
15	10-Apr - 16-Apr 22	4593	119063
16	17-Apr - 23-Apr 22	3553	122616
17	24-Apr - 30-Apr 22	1854	124470
18	1-May - 7-May 22		
19	8-May - 14-May 22	485	125763
20	15-May - 21-May 22	402	126165
21	22-May - 28-May 22	337	126502
22	29-May - 4-Jun 22		
23	5-Jun - 11-Jun 22		
24	12-Jun - 18-Jun 22	47	127354
25	19-Jun - 25-Jun 22	30	127384
26	26-Jun - 2-Jul 22		
27	3-Jul - 9-Jul 22		
28	10-Jul - 16-Jul 22	68	127569
29	17-Jul - 23-Jul 22	150	127719
30	24-Jul - 30-Jul 22	147	127866
31	31-Jul - 6-Aug 22		
32	7-Aug - 13-Aug 22	122	128113
33	14-Aug - 20-Aug 22	116	128229
34	21-Aug - 27-Aug 22	55	128284
35	28-Aug - 3-Sep 22		
36	4-Sep - 10-Sep 22		
37	11-Sep - 17-Sep 22	23	128465
38	18-Sep - 24-Sep 22	41	128506
39	25-Sep - 1-Oct 22		
40	2-Oct - 8-Oct 22		
41	9-Oct - 15-Oct 22	20	128586
42	16-Oct - 22-Oct 22	25	128611
43	23-Oct - 29-Oct 22	35	128646
44	30-Oct - 5-Nov 22		
45	6-Nov - 12-Nov 22		
46	13-Nov - 19-Nov 22	72	128840
47	20-Nov - 26-Nov 22	120	128960
48	27-Nov - 3-Dec 22		
49	4-Dec - 10-Dec 22		
50	11-Dec - 17-Dec 22	78	129224
51	18-Dec - 24-Dec 22	20	129244
52	25-Dec - 31-Dec 22	48	129292
53	1-Jan - 7-Jan 23	8	129761

Year / Week	2023		
	Period	Case number	Accumulate case
1	1-Jan - 7-Jan 23	8	129761
2	8-Jan - 14-Jan 23		
3	15-Jan - 21-Jan 23	9	129314
4	22-Jan - 28-Jan 23	3	129317
5	29-Jan - 4-Feb 23		
6	5-Feb - 11-Feb 23		
7	12-Feb - 18-Feb 23	5	129324
8	19-Feb - 25-Feb 23	0	129324
9	26-Feb - 4-Mar 23		
10	5-Mar - 11-Mar 23		
11	12-Mar - 18-Mar 23		
12	19-Mar - 25-Mar 23	2	129326
13	26-Mar - 1-Apr 23		
14	2-Apr - 8-Apr 23		
15	9-Apr - 15-Apr 23	2	129334
16	16-Apr - 22-Apr 23	7	129341
17	23-Apr - 29-Apr 23	27	129368
18	30-Apr - 6-May 23		
19	7-May - 13-May 23	80	129488
20	14-May - 20-May 23	58	129546
21	21-May - 27-May 23	44	129590
22	28-May - 3-Jun 23		
23	4-Jun - 10-Jun 23		
24	11-Jun - 17-Jun 23	37	129738
25	18-Jun - 24-Jun 23	15	129753
26	25-Jun - 1-Jul 23	6	129298
27	2-Jul - 8-Jul 23		
28	9-Jul - 15-Jul 23	3	129231
29	16-Jul - 22-Jul 23	4	129778
30	23-Jul - 29-Jul 23	2	129780
31	30-Jul - 5-Aug 23		
32	6-Aug - 12-Aug 23		
33	13-Aug - 19-Aug 23		
34	20-Aug - 26-Aug 23	2	129782
35	27-Aug - 2-Sep 23		
36	3-Sep - 9-Sep 23	7	129789
37	10-Sep - 16-Sep 23		
38	17-Sep - 23-Sep 23		
39	24-Sep - 30-Sep 23	4	129793
40	1-Oct - 7-Oct 23		
41	8-Oct - 14-Oct 23		
42	15-Oct - 21-Oct 23		
43	22-Oct - 28-Oct 23		
44	29-Oct - 4-Nov 23		
45	5-Nov - 11-Nov 23	2	129797
46	12-Nov - 18-Nov 23	4	129801
47	19-Nov - 25-Nov 23	7	129808
48	26-Nov - 2-Dec 23		
49	3-Dec - 9-Dec 23		
50	10-Dec - 16-Dec 23	7	129839
51	17-Dec - 23-Dec 23	20	129859
52	24-Dec - 30-Dec 23	19	129878
53	31-Dec - 6-Jan 24	62	130319

Week \ Year	2024		
	Period	Case number	Accumulate case
1	7-Jan - 13-Jan 24	4	129900
2	14-Jan - 20-Jan 24	20	129920
3	21-Jan - 27-Jan 24	11	129931
4	28-Jan - 3-Feb 24	14	129945
5	4-Feb - 10-Feb 24		
6	11-Feb - 17-Feb 24	13	129979
7	18-Feb - 24-Feb 24	14	129993
8	25-Feb - 2-Mar 24	8	130001
9	3-Mar - 9-Mar 24		
10	10-Mar - 16-Mar 24	5	130010
11	17-Mar - 23-Mar 24	8	130018
12	24-Mar - 30-Mar 24	6	130024
13	31-Mar - 6-Apr 24		
14	7-Apr - 13-Apr 24	13	130042
15	14-Apr - 20-Apr 24	13	130055
16	21-Apr - 27-Apr 24	13	130068
17	28-Apr - 4-May 24		
18	5-May - 11-May 24		
19	12-May - 18-May 24	51	130194
20	19-May - 25-May 24	63	130257
21	26-May - 1-Jun 24		
22	2-Jun - 8-Jun 24		
23	9-Jun - 15-Jun 24	41	130412
24	16-Jun - 22-Jun 24		
25	23-Jun - 29-Jun 24		
26	30-Jun - 6-Jul 24		
27	7-Jul - 13-Jul 24		
28	14-Jul - 20-Jul 24		
29	21-Jul - 27-Jul 24		
30	28-Jul - 3-Aug 24		

Number of CIFs During COVID-19 Pandemin in Nakhon Si Thammarat 2021

Month	CIFs
1	0
2	0
3	0
4	8
5	9
6	21
7	36
8	45
9	47
10	56
11	57
12	57

Preparedness for establishment Raw data

ID	Loca 1	Loca 2	Loca 3	Loca 4	Loca 5	Loca 6	Loca 7	Loca 8	Loca 9	Loca 10	Staf 1	Sup 1	Sup 2	Sup 3
A1	1	1	0	1	1	1	0	0	1	1	1	1	1	1
A2	1	1	1	1	1	1	1	0	1	1	1	1	1	1
A3	1	1	0	1	0	1	1	0	1	1	1	1	1	1
A4	1	1	0	1	1	1	1	1	1	1	1	1	1	1
A5	0	1	1	1	1	1	1	1	1	0	1	1	1	1
A6	1	1	0	1	1	1	1	0	1	1	1	1	1	1
A7	1	1	0	1	0	1	1	0	1	1	0	1	1	1
A8	1	1	0	1	1	1	1	1	1	1	0	1	1	1
A9	1	1	1	1	1	1	1	0	1	1	1	1	1	1
A10	0	1	0	1	1	1	1	0	1	1	0	1	1	1
A11	1	1	1	1	1	1	0	0	1	1	0	0	1	1
A12	1	1	0	1	1	1	0	0	1	1	0	1	1	1
A13	1	1	0	1	1	1	0	0	1	1	1	1	1	1
A14	1	1	0	1	1	1	1	0	1	1	0	1	1	1
A15	1	1	1	1	1	1	1	0	1	1	0	1	1	1
A16	1	1	0	1	1	1	1	0	1	1	1	1	1	1
A17	1	1	0	1	1	1	0	0	1	1	1	1	1	1
A18	1	1	0	1	1	1	1	0	0	1	0	1	1	1
A19	1	1	0	1	1	1	1	1	1	1	1	1	1	1
A20	1	1	1	1	1	1	1	1	1	1	1	1	1	1
A21	1	1	0	1	0	1	0	0	1	1	1	1	1	1
A22	1	1	0	1	1	1	1	0	0	1	0	1	1	1
A23	1	1	1	1	1	1	1	1	1	1	1	1	1	1
A24	1	1	0	1	1	1	0	0	1	1	0	1	1	1
A25	1	1	0	1	1	1	1	0	1	1	1	1	1	1
A26	1	1	0	1	1	1	1	0	1	1	1	1	1	1
A27	1	1	1	1	1	1	1	1	1	1	1	1	1	1
A28	1	1	0	0	1	1	0	0	0	1	0	0	1	1
A29	1	1	0	1	1	1	1	0	1	1	0	1	1	1

ID	Loca 1	Loca 2	Loca 3	Loca 4	Loca 5	Loca 6	Loca 7	Loca 8	Loca 9	Loca 10	Staf 1	Sup 1	Sup 2	Sup 3
A30	1	1	0	1	1	1	1	0	1	1	1	1	1	1
A31	1	1	1	1	1	1	1	1	1	1	1	1	1	1
A32	1	1	1	1	1	1	1	1	1	1	0	1	1	1
A33	1	1	0	1	1	1	1	0	1	1	1	1	1	1
A34	1	1	0	1	1	1	1	0	1	1	0	1	1	1
A35	1	1	1	1	1	1	1	1	1	1	1	1	1	1
A36	1	1	0	0	1	1	0	0	1	1	1	1	1	1
A37	1	1	0	1	1	1	1	0	0	1	0	1	1	1
A38	1	1	0	1	0	1	0	1	1	1	0	1	1	1
A39	1	1	0	1	1	1	1	0	1	1	0	1	1	1
A40	1	1	0	1	1	1	0	0	1	1	1	1	1	1
A41	1	1	0	1	1	1	1	0	1	1	1	1	1	1
A42	1	1	0	0	0	1	0	0	1	1	1	1	1	1
A43	1	1	1	1	1	1	1	1	1	1	1	1	1	1
A44	1	1	0	1	1	1	1	0	1	1	1	1	1	1
A45	1	1	0	1	0	1	1	1	1	1	1	1	1	1
A46	1	1	1	1	1	1	1	1	1	1	1	1	1	1
A47	1	1	1	1	1	1	0	0	1	1	0	1	1	1
A48	1	1	0	1	1	1	1	0	1	1	1	1	1	1
A49	1	1	0	1	1	1	0	0	1	1	1	1	1	1
A50	0	0	0	1	1	1	1	1	1	1	1	1	1	1
A51	1	1	0	1	1	1	0	0	1	1	1	1	1	1
A52	1	1	0	1	1	1	1	0	1	1	1	1	1	1
A53	1	1	1	1	0	1	1	1	1	1	1	1	1	1
A54	1	1	1	1	1	1	1	1	1	1	1	1	1	1
A55	1	1	1	1	0	1	1	1	1	1	1	1	1	1
A56	1	1	1	1	1	1	0	0	1	1	1	1	1	1
A57	1	1	1	1	1	1	1	1	1	1	1	1	1	1

ID	Sup 4	Sup 5	Gen 1	Gen 2	Gen 3	Gen 4	Gen 5	Gen 6	Gen 7	Gen 8	Gen 9	Gen 10	Gen 11	Gen 12
A1	1	1	1	0	0	1	1	1	1	1	1	0	1	1
A2	1	1	1	1	1	1	1	1	1	1	1	1	1	1
A3	1	1	1	0	1	1	0	1	0	1	0	0	1	1
A4	1	1	1	1	1	1	1	1	1	1	1	1	1	1
A5	1	1	1	1	1	1	1	1	1	1	1	1	1	1
A6	1	1	1	1	1	1	1	1	0	1	1	1	1	1
A7	1	1	1	1	1	1	0	1	0	1	1	1	1	1
A8	1	1	1	1	1	1	1	1	1	1	1	1	1	1
A9	1	1	1	1	1	1	1	1	1	1	1	1	1	1
A10	1	1	1	1	1	1	0	1	0	1	1	1	1	1
A11	1	1	1	0	1	1	0	0	0	1	1	1	0	1
A12	1	1	1	1	1	1	0	1	0	0	1	1	1	1
A13	1	1	1	1	1	1	1	1	1	1	1	1	1	1
A14	1	1	1	0	0	1	1	1	0	1	1	1	1	1
A15	1	1	1	1	1	1	1	1	1	1	1	1	1	1
A16	1	1	1	1	1	1	1	1	0	1	1	1	1	1
A17	1	1	1	1	1	1	1	1	0	1	1	1	1	1
A18	1	1	1	1	0	1	0	0	0	1	1	1	1	1
A19	1	1	1	1	1	1	1	1	1	1	1	1	1	1
A20	1	1	1	1	1	1	1	1	1	1	1	1	1	1
A21	1	1	1	1	1	1	0	1	0	1	1	1	1	1
A22	1	1	1	1	0	1	0	1	0	1	1	1	1	1
A23	1	1	1	1	1	1	1	1	1	1	1	1	1	1
A24	1	1	0	0	1	1	1	1	1	1	1	1	1	1
A25	1	1	1	1	1	1	1	1	1	1	1	1	1	1
A26	1	1	1	1	1	1	1	1	1	1	1	1	1	1
A27	1	1	1	1	1	1	1	1	1	1	1	1	1	1
A28	1	1	1	1	0	0	0	1	1	1	1	1	1	1
A29	1	1	1	1	1	1	1	1	1	1	1	1	1	1

ID	Sup 4	Sup 5	Gen 1	Gen 2	Gen 3	Gen 4	Gen 5	Gen 6	Gen 7	Gen 8	Gen 9	Gen 10	Gen 11	Gen 12
A30	1	1	1	1	1	1	1	1	1	1	1	1	1	1
A31	1	1	1	1	1	1	1	1	1	1	1	1	1	1
A32	1	1	1	1	1	1	0	1	1	1	1	0	1	1
A33	1	1	1	1	1	1	1	1	1	1	1	1	1	1
A34	1	1	1	1	1	1	1	1	1	1	1	1	1	1
A35	1	1	1	1	1	1	1	1	1	1	1	1	1	1
A36	1	1	1	1	1	1	1	1	1	1	1	1	1	1
A37	1	1	1	1	0	1	0	1	0	1	1	1	1	1
A38	1	1	1	1	1	1	0	1	1	0	0	0	1	1
A39	1	1	1	1	1	1	1	1	1	1	1	1	1	1
A40	1	1	1	1	1	1	1	1	1	1	1	1	1	1
A41	1	1	1	1	1	1	1	1	1	1	1	1	1	1
A42	1	1	1	1	1	1	1	1	0	1	0	0	1	1
A43	1	1	1	1	1	1	1	1	1	1	1	1	1	1
A44	1	1	1	1	1	1	1	1	0	1	1	1	1	1
A45	1	1	1	0	1	1	1	1	0	1	1	1	1	1
A46	1	1	1	1	1	1	1	1	1	1	1	1	1	1
A47	1	1	1	1	1	1	0	1	0	1	0	0	1	1
A48	1	1	1	1	1	1	1	1	1	1	1	1	1	1
A49	1	1	1	1	1	1	0	1	0	1	1	0	1	1
A50	1	1	1	0	1	1	1	1	0	1	1	1	1	1
A51	1	1	1	1	1	1	1	1	0	1	1	1	1	1
A52	1	1	1	1	1	1	1	1	1	1	1	1	1	1
A53	1	1	1	1	1	1	1	1	0	1	1	1	1	1
A54	1	1	1	1	1	1	0	1	1	1	1	1	1	1
A55	1	1	1	1	1	1	1	1	0	1	1	1	1	1
A56	1	1	1	1	1	1	1	1	1	1	1	1	1	1
A57	1	1	1	1	1	1	1	1	1	1	1	1	1	1

ID	Gen 13	Gen 14	Gen 15	ENH 1	ENH 2	ENH 3	ENH 4	ENH 5	ENH 6	ENH 7	ENH 8
A1	0	0	1	1	1	1	1	1	1	1	1
A2	1	0	1	1	1	1	1	1	1	1	1
A3	0	0	1	1	1	0	1	1	1	0	1
A4	1	1	1	1	1	1	1	1	1	1	1
A5	1	0	1	1	1	1	1	1	1	1	1
A6	1	1	1	1	1	1	1	1	1	1	1
A7	0	1	1	1	1	0	1	1	0	1	0
A8	1	1	1	1	1	1	1	1	1	1	1
A9	1	0	1	1	1	1	1	1	1	1	1
A10	0	0	1	1	1	0	1	1	0	1	1
A11	0	0	0	1	1	0	1	1	1	1	1
A12	1	0	0	1	1	0	1	1	0	1	1
A13	1	1	1	1	1	1	1	1	1	1	1
A14	0	1	1	1	1	1	1	1	0	1	1
A15	1	1	1	1	1	1	1	1	1	1	1
A16	0	0	1	1	1	1	1	1	1	1	1
A17	0	0	1	1	1	1	1	1	1	1	1
A18	0	0	0	1	1	1	1	1	1	1	0
A19	1	1	1	1	1	1	1	1	1	1	1
A20	1	0	0	1	1	1	1	1	1	1	1
A21	0	0	0	1	1	1	1	1	1	1	1
A22	1	0	0	1	1	1	1	1	0	1	0
A23	1	1	1	1	1	1	1	1	1	1	1
A24	0	0	0	1	1	0	1	1	0	1	0
A25	0	1	1	1	1	1	1	1	0	1	0
A26	0	1	1	1	1	1	1	1	1	1	1
A27	1	1	1	1	1	1	1	1	1	1	1
A28	0	1	0	1	1	0	1	1	0	1	0
A29	1	1	1	1	1	1	1	1	0	1	1

ID	Gen 13	Gen 14	Gen 15	ENH 1	ENH 2	ENH 3	ENH 4	ENH 5	ENH 6	ENH 7	ENH 8
A30	1	1	1	1	1	1	1	1	1	1	1
A31	1	1	1	1	1	1	1	1	1	1	1
A32	0	0	0	1	1	1	1	1	1	1	1
A33	1	1	1	1	1	1	1	1	1	1	1
A34	0	1	0	1	1	1	1	1	1	1	1
A35	1	1	1	1	1	1	1	1	1	1	1
A36	0	1	1	1	1	1	1	0	1	1	1
A37	1	0	0	1	1	1	1	1	0	1	0
A38	1	0	0	0	1	0	1	1	0	0	1
A39	0	1	1	1	1	1	1	1	1	1	1
A40	0	1	1	1	1	1	1	1	1	1	1
A41	0	1	1	1	1	1	1	1	1	1	1
A42	0	1	1	1	1	1	1	1	1	1	1
A43	0	0	1	1	1	1	1	1	1	1	1
A44	0	0	0	1	1	1	1	1	1	1	1
A45	0	1	1	1	1	1	1	1	1	1	1
A46	1	1	1	1	1	1	1	1	1	1	1
A47	0	0	1	1	1	1	1	1	0	1	1
A48	0	0	1	1	1	1	1	1	1	1	1
A49	0	1	1	1	1	1	1	1	1	1	1
A50	1	1	1	1	1	0	1	1	1	1	1
A51	0	1	1	1	1	0	1	1	1	1	1
A52	0	1	1	1	1	1	1	1	1	1	1
A53	0	1	1	1	1	1	1	1	0	1	1
A54	0	0	0	1	1	1	1	1	1	1	1
A55	0	1	1	1	1	1	1	1	0	1	1
A56	0	1	1	1	1	1	1	1	1	1	1
A57	1	1	1	1	1	1	1	1	1	1	1

Environmental health implementation Raw data

ID	B1	B2	B3	B4	B5	B6	B7	B8	B9	B10	B11	B12	B13	B14
A1	NA	NA	NA	NA	2	2	1	2	2	2	2	2	2	2
A2	2	2	2	2	1	2	1	2	2	2	2	2	2	1
A3	NA	NA	NA	NA	2	2	1	2	2	2	2	2	2	1
A4	NA	NA	NA	NA	1	2	1	2	2	2	2	2	2	1
A5	NA	NA	NA	NA	2	2	2	2	2	2	2	2	2	2
A6	NA	NA	NA	NA	2	2	2	1	2	2	1	2	2	2
A7	NA	NA	NA	NA	0	2	2	2	2	2	2	2	2	2
A8	NA	NA	NA	NA	2	2	2	2	2	2	2	2	2	2
A9	NA	NA	NA	NA	2	2	2	2	2	2	2	2	2	2
A10	NA	NA	NA	NA	1	2	1	1	2	2	2	2	2	2
A11	NA	NA	NA	NA	1	2	1	2	2	2	2	2	2	2
A12	1	1	2	2	1	2	2	2	2	2	2	2	2	2
A13	0	0	0	0	2	2	2	2	2	2	2	2	2	2
A14	NA	NA	NA	NA	1	2	1	1	2	2	1	2	2	1
A15	NA	NA	NA	NA	1	2	0	2	2	2	2	2	2	2
A16	NA	NA	NA	NA	2	2	1	2	2	2	2	2	2	2
A17	NA	NA	NA	NA	1	2	1	2	2	2	1	2	2	1
A18	1	1	2	2	1	2	1	1	2	2	2	2	2	1
A19	2	2	2	2	1	2	2	2	2	2	2	2	2	2
A20	NA	NA	NA	NA	2	2	1	2	2	2	2	2	2	2
A21	NA	NA	NA	NA	1	1	1	2	2	2	2	2	2	1
A22	1	1	2	2	1	2	1	1	2	2	2	2	2	1
A23	NA	NA	NA	NA	2	2	2	2	2	2	2	2	2	2
A24	NA	NA	NA	NA	1	1	1	2	2	2	2	2	2	1
A25	NA	NA	NA	NA	1	2	1	2	2	2	2	2	2	2
A26	NA	NA	NA	NA	2	2	1	2	2	2	2	2	2	2
A27	NA	NA	NA	NA	1	2	1	2	2	2	2	2	2	2
A28	NA	NA	NA	NA	2	2	1	2	2	2	1	2	2	2

ID	B1	B2	B3	B4	B5	B6	B7	B8	B9	B10	B11	B12	B13	B14
A29	NA	NA	NA	NA	1	2	1	2	2	2	2	2	2	1
A30	NA	NA	NA	NA	2	2	2	1	2	2	1	2	2	2
A31	NA	NA	NA	NA	2	2	2	2	2	2	2	2	2	2
A32	NA	NA	NA	NA	1	2	0	2	2	2	2	2	2	2
A33	NA	NA	NA	NA	2	2	1	2	2	2	2	2	2	2
A34	NA	NA	NA	NA	1	2	1	2	2	2	2	2	2	2
A35	NA	NA	NA	NA	2	2	2	2	2	2	2	2	2	2
A36	NA	NA	NA	NA	2	2	2	2	2	2	2	2	2	2
A37	NA	NA	NA	NA	1	2	1	2	2	2	2	2	2	1
A38	NA	NA	NA	NA	1	2	2	2	2	2	2	2	2	2
A39	NA	NA	NA	NA	2	2	1	2	2	2	2	2	2	2
A40	NA	NA	NA	NA	1	2	2	2	2	2	2	2	2	2
A41	NA	NA	NA	NA	1	1	1	2	1	1	2	1	1	1
A42	NA	NA	NA	NA	2	2	1	2	2	2	2	2	2	2
A43	NA	NA	NA	NA	2	2	1	2	2	2	2	2	2	2
A44	NA	NA	NA	NA	1	2	1	2	2	2	2	2	1	1
A45	NA	NA	NA	NA	2	2	1	2	2	2	2	2	2	2
A46	2	2	2	2	2	2	2	2	2	2	2	2	2	2
A47	NA	NA	NA	NA	1	2	2	2	2	2	2	2	2	2
A48	NA	NA	NA	NA	1	2	1	2	2	2	2	2	2	2
A49	NA	NA	NA	NA	2	2	2	2	2	2	0	2	2	2
A50	NA	NA	NA	NA	1	2	2	2	2	2	2	2	2	2
A51	NA	NA	NA	NA	1	2	2	2	2	2	2	2	2	2
A52	NA	NA	NA	NA	1	2	1	2	2	2	2	2	2	2
A53	NA	NA	NA	NA	2	2	2	2	2	2	2	0	2	2
A54	NA	NA	NA	NA	2	2	2	2	2	2	2	2	2	1
A55	NA	NA	NA	NA	2	2	2	2	2	2	2	1	2	2
A56	NA	NA	NA	NA	2	2	2	2	2	2	2	2	2	2
A57	NA	NA	NA	NA	2	2	1	2	2	2	2	2	2	1

Remark NA refers to not available, as there was no operation in the CIF related to that item in the questionnaire.

ID	B15	B16	B17	B18	B19	B20	B21	B22	B23	B24	B25	B26	B27	B28
A1	2	2	2	NA	NA	NA	1	0	2	NA	NA	2	2	2
A2	2	2	2	2	NA	NA	1	1	2	NA	NA	2	2	2
A3	2	2	2	0	NA	NA	1	0	2	NA	NA	2	2	2
A4	1	2	2	1	0	0	1	0	2	NA	NA	2	2	2
A5	1	2	2	2	2	1	2	1	2	NA	NA	2	2	2
A6	1	2	2	1	NA	NA	1	0	2	NA	NA	2	2	2
A7	2	2	2	2	NA	NA	2	0	2	NA	NA	2	2	2
A8	2	2	2	2	1	2	1	2	2	NA	NA	2	2	2
A9	1	2	2	2	NA	NA	2	1	2	NA	NA	1	1	1
A10	1	2	1	2	NA	NA	2	0	2	NA	NA	2	1	2
A11	2	2	2	NA	NA	NA	2	0	2	NA	NA	2	2	2
A12	2	2	2	NA	NA	NA	0	0	2	NA	NA	1	2	1
A13	2	2	2	NA	NA	NA	2	0	2	NA	NA	2	2	2
A14	1	2	1	2	NA	NA	1	0	2	NA	NA	1	2	2
A15	2	2	2	2	NA	NA	2	2	2	NA	NA	2	2	2
A16	2	2	2	2	NA	NA	2	0	2	NA	NA	2	2	2
A17	2	2	1	NA	NA	NA	1	0	2	NA	NA	1	1	2
A18	2	2	2	1	NA	NA	1	0	2	NA	NA	2	2	2
A19	2	2	2	2	0	0	2	1	2	NA	NA	2	2	2
A20	2	2	2	2	2	2	2	1	2	NA	NA	2	2	2
A21	2	2	2	NA	NA	NA	0	0	2	NA	NA	2	1	2
A22	2	2	1	1	NA	NA	1	0	2	NA	NA	2	2	2
A23	2	2	2	2	2	1	2	2	2	NA	NA	2	2	2
A24	2	2	2	NA	NA	NA	2	0	2	NA	NA	2	2	2
A25	2	2	2	2	NA	NA	2	0	2	NA	NA	2	2	2
A26	2	2	2	2	NA	NA	2	1	2	NA	NA	2	2	2
A27	2	2	2	2	1	0	1	0	2	NA	NA	2	2	2
A28	2	2	2	NA	NA	NA	0	0	2	NA	NA	2	2	2

ID	B15	B16	B17	B18	B19	B20	B21	B22	B23	B24	B25	B26	B27	B28
A29	1	2	2	1	NA	NA	1	0	2	NA	NA	2	2	2
A30	1	2	2	1	NA	NA	1	0	2	NA	NA	2	2	2
A31	2	2	2	2	0	0	2	0	2	NA	NA	2	2	2
A32	2	2	2	2	2	1	2	2	2	NA	NA	2	2	2
A33	2	2	2	2	NA	NA	2	1	2	NA	NA	2	2	2
A34	2	2	2	2	NA	NA	1	0	2	NA	NA	2	2	2
A35	2	2	2	2	0	0	1	0	2	2	2	2	2	2
A36	2	2	2	NA	NA	NA	2	0	2	NA	NA	2	2	2
A37	2	2	2	2	NA	NA	2	0	2	NA	NA	2	1	2
A38	1	2	2	NA	0	0	1	0	2	NA	NA	2	2	2
A39	2	2	2	1	NA	NA	2	0	2	NA	NA	2	2	2
A40	2	2	2	NA	NA	NA	0	0	2	1	2	2	2	2
A41	2	1	1	2	NA	NA	2	0	1	NA	Na	1	1	1
A42	2	2	2	NA	NA	NA	2	0	2	NA	Na	2	2	2
A43	2	2	2	0	0	0	0	0	2	2	0	2	1	1
A44	2	1	2	1	NA	NA	2	0	2	NA	NA	2	2	2
A45	2	2	2	1	0	0	1	0	2	NA	NA	2	2	2
A46	2	2	2	2	2	2	2	2	2	NA	NA	2	2	2
A47	2	2	2	NA	NA	NA	1	0	2	NA	NA	2	2	2
A48	2	2	2	1	NA	NA	1	0	2	NA	NA	2	2	2
A49	2	2	2	NA	NA	NA	2	0	2	NA	NA	2	2	2
A50	2	2	2	1	0	0	2	0	2	NA	NA	2	2	2
A51	2	2	2	NA	NA	NA	0	0	2	NA	NA	2	2	2
A52	2	0	2	1	NA	NA	2	0	0	2	1	2	2	2
A53	2	2	2	0	0	0	0	0	2	NA	NA	2	2	2
A54	2	2	2	0	0	0	0	0	2	NA	NA	2	2	2
A55	2	2	2	0	0	0	0	0	2	NA	NA	2	2	2
A56	2	2	2	NA	NA	NA	2	2	2	NA	NA	2	2	2
A57	2	2	2	2	0	0	0	0	2	NA	NA	1	1	1

Remark NA refers to not available, as there was no operation in the CIF related to that item in the questionnaire.

ID	B29	B30	B31	B32	B33	B34	B35	B36
A1	2	2	2	2	2	2	2	2
A2	2	2	2	2	2	2	2	2
A3	2	2	2	2	2	2	2	2
A4	2	2	1	2	2	2	2	2
A5	2	2	2	2	2	2	2	2
A6	2	2	2	2	2	2	2	1
A7	2	2	2	2	2	2	2	2
A8	2	2	2	2	2	2	2	2
A9	2	2	2	2	2	2	2	2
A10	2	2	2	2	2	2	2	2
A11	1	2	2	2	2	2	2	2
A12	2	2	2	2	2	2	2	1
A13	2	2	2	2	2	2	2	2
A14	1	2	2	2	2	2	2	1
A15	2	2	2	2	2	2	2	2
A16	2	1	2	2	2	2	2	2
A17	1	1	2	2	2	2	2	1
A18	1	2	2	2	2	2	2	1
A19	2	2	2	2	2	2	2	2
A20	2	2	2	2	2	2	2	1
A21	1	2	2	2	2	2	2	1
A22	2	2	2	2	2	2	2	1
A23	2	2	2	2	2	2	2	2
A24	1	2	2	2	2	2	2	1
A25	2	2	2	2	2	2	2	1
A26	2	2	2	2	2	2	2	2
A27	2	2	2	2	2	2	2	1
A28	2	2	2	2	2	2	2	1

ID	B29	B30	B31	B32	B33	B34	B35	B36
A29	2	2	2	2	2	2	2	2
A30	2	2	2	2	2	2	2	2
A31	2	2	2	2	2	2	2	1
A32	2	2	2	2	2	2	2	2
A33	2	2	2	2	2	2	2	1
A34	2	2	2	2	2	2	2	2
A35	2	2	2	2	2	2	2	1
A36	2	2	2	2	2	2	2	2
A37	1	2	2	2	2	2	2	1
A38	1	1	2	2	2	2	2	1
A39	2	2	2	2	2	2	2	1
A40	2	2	2	2	2	2	2	2
A41	1	1	1	1	1	1	1	1
A42	2	2	2	2	2	2	2	0
A43	2	2	2	2	2	2	2	2
A44	2	2	2	2	2	2	2	1
A45	2	2	2	2	2	2	2	2
A46	2	2	2	2	2	2	2	2
A47	2	2	2	2	2	2	2	1
A48	2	2	2	2	2	2	2	1
A49	2	2	2	2	2	2	2	2
A50	2	2	2	2	2	2	2	2
A51	2	2	2	2	2	2	2	2
A52	1	2	2	2	2	2	2	2
A53	2	2	2	2	2	2	2	2
A54	2	2	2	2	2	2	2	2
A55	2	2	2	2	2	2	2	2
A56	2	2	2	2	2	2	2	2
A57	1	1	1	1	1	1	1	2

Remark NA refers to not available, as there was no operation in the CIF related to that item in the questionnaire.
