# Supplementary material for: Environmental health management in local community isolation facilities during COVID-19 pandemic: A case study in Nakhon Si Thammarat, Thailand
Source: PLoS One. 2025 Oct 6;20(10):e0333638. doi: 10.1371/journal.pone.0333638 (PMC12500105; doi:10.1371/journal.pone.0333638)
Supplement: Attachment — Submitted filename: PONE-D-25-02239-Response to Reviewers.pdf [file pone.0333638.s004.pdf]

Dear Dr. Babak Pakbin,

Thank you for giving me the opportunity to submit a revised draft of my manuscript titled "Environmental health management in local community isolation facilities during COVID-19 pandemic: A case study in Nakhon Si Thammarat, Thailand" to *PLOS ONE*. I appreciate the time and effort you and the reviewers have dedicated to providing valuable feedback on the manuscript. I am grateful to the reviewers for their insightful comments on my paper. I have incorporated changes to reflect most of the suggestions provided by the reviewers. The manuscript's changes were shown using track changes in the separated file (File name: PONE-D-25-02239-Revised Manuscript with Track Changes). Point-by-point responses to the reviewer's comments and concerns are presented as follows. **For your information, the line numbers mentioned in this document refer to the lines in the file with track change, not the unmarked version of the revised manuscript**.

Comments from Reviewer 1

1. Abstract

- ***Comment 1.1****: Specify the time period of the study.*

  Response: We have added the following information (underlined) to a sentence in the abstract.

  Line 31 – 32: "Two questionnaires were collected from the founding and operating staff from 27 May to 5 October 2022."

- ***Comment 1.2****: Clarify the total number of staff enrolled.*

  Response: We have added the following information (underlined) to a sentence in the abstract.

  Line 30 – 31: "A total number of 114 staff working during the establishment and operation of 57 CIFs were enrolled."

- ***Comment 1.3***: *Brief mention of the statistical methods used.*

  Response: We have added the following information (underlined) to a sentence in the abstract.

  Line 35 – 36: "The data was presented as the levels of preparedness and implementation of CIFs and analyzed by descriptive statistics."

- ***Comment 1.4***: *Consider adding a brief background sentence.*

  Response: Thank you for this suggestion. The brief background, "Community isolation facilities (CIFs) were adopted as a containment strategy in many countries during the COVID-19 pandemic, but information on environmental health management in CIFs is unknown," is already included in the first sentence of the abstract. We added the following sentence to the abstract to provide additional information on the rationale for choosing the study location.

  Line 28 – 30: "The study was conducted in Nakhon Si Thammarat Province, Thailand, which had the second-highest number of COVID-19 patients in the country, following Bangkok, at the time of the study."

- ***Comment 1.5***: *The final statement suggests that guidelines should be adopted for future disease outbreaks but doesn't specify how they should be improved.*

  Response: Thank you for pointing this out. The guidelines should be adopted to be practical and based on the limited availability of resources and local constraints in each country during emergencies while still being effective in preventing the spread of diseases. Therefore, a phrase has been briefly added, as shown in the following sentence.

  Line 44 – 63: "The guidelines by the Department of Health suggested as fundamental for environmental health management of CIFs in this study should be appropriately adopted based on the different contexts of each country to ensure preparedness for future infectious disease outbreaks."

- ***Comment 1.6:*** *Provide more information about questionnaire.*

  Response: We have added the following information (underlined) to the abstract.

  Line 31 - 34: "<u>Two questionnaires were collected from the founding and operating staff</u> from 27 May to 5 October 2022. One questionnaire, designed based on the guidelines for establishing CIFs developed by the Department of Health, <u>was used to examine the preparedness of the founding staff. Another questionnaire was developed to investigate the implementation by the operating staff</u>."

2. Introduction

- ***Comment 2.1****: The research gap is stated but could be emphasized more clearly.*

  Response: Thank you for this suggestion. We have added the following sentences to emphasize the research gaps and benefits of the study.

  Line 111 - 113: "Until now, however, no research has comprehensively studied environmental health issues related to the COVID-19 pandemic. Holistic environmental health management would be beneficial for effectively reducing the spread of COVID-19 infections."

  Line 114 – 115: "Exploring this information is essential to ensure the success of CIFs' operation."

- ***Comment 2.2****: The objectives should be stated more clearly and concisely.*

  Response: Agree. We decided to modify the objective to make it more concise as follows.

  Line 116 – 118: "This retrospective research aimed to study the preparedness and environmental health implementation in local CIFs in Nakhon Si Thammarat Province, Thailand, during the COVID-19 pandemic."

3. Method

- ***Comment 3.1***: *Clarify the sampling method.*

  Response: There was no sampling in this study. During the study period, all 114 CIFs in the province. However, only 57 CIFs met the criteria and consented to participate in the study. Therefore, the number 57, half of the total number of CIFs (114 CIFs), is unrelated to the sampling process. We have added the following sentences to clarify this point.

  Line 148 – 150: "During the study period, there were 114 CIFs in the province. Out of these, 57 CIFs met the criteria and consented to participate in the study. Accordingly, 57 founding and 57 operating staff members were enrolled in the study."

- ***Comment 3.2***: *Provide more details on how the 57 CIFs were selected from the total 114.*

  Response: As described in response to comment 3.1, there was no selection or sampling. All CIFs that met the criteria and consented to participate in the study were included.

- ***Comment 3.3***: *Provide the process of developing questionnaires based on guidelines.*

  Response: Thank you for this suggestion. We have added the following sentences to elaborate on the questionnaire development process.

  Line 190 – 206: "The checklist was designed based on the guidelines for establishing CIFs the Department of Health developed. Out of the seven elements in the guideline for CIF preparedness, four were included: 1) administration, 2) location setting and infrastructure, 3) food supply, and 4) infectious waste management and sanitation. The three excluded elements included general facilities and medical equipment, health promotion activities for patients, and surveillance systems. However, specific issues related to surveillance systems, such as wastewater and excreta management, were included in the checklist. Some elements were excluded to align the checklist with the study's

objectives and make it more concise for data collection. Additionally, some issues in the checklist, which could have led to uncertainty in judgment by the participants, were modified by adding reference values, such as the adequacy of toilets. Moreover, other issues were added to ensure the checklist had more comprehensive content regarding environmental health management."

- ***Comment 3.4***: *Provide rationale for the specific score ranges and cut-off points.*

Response: Thank you for pointing this out. The specific score ranges were calculated based on Bloom's cut-off categories: poor (less than 60%), moderate (60 – 79%), and good (80 – 100%) for levels of preparedness and implementation (all dimensions). To specify the score ranges, we need to mention every dimension of preparedness (4 dimensions) and implementation (6 dimensions), but this might be too lengthy. The cut-off presented in the Data Analysis is sufficient to demonstrate how we interpret the scores for the levels of preparedness and implementation, as shown in Tables 4 and 6, respectively. Therefore, we decided to delete the score ranges.

"The score ranges of the poor, moderate, and good levels were 0 – 14.39, 14.40 – 19.19, and 19.20 – 24.00, respectively." was deleted.

"The score ranges of the poor, moderate, and good levels were 0 – 32.39, 32.40 – 43.19, and 43.20 – 54.00, respectively." was deleted.

For the cut-off point, we used Bloom's cut-off categories: poor (less than 60%), moderate (60 – 79%), and good (80 – 100%). We have added two references for the cut-off point, which are as follows. The references were also added to the reference list.

Line 228 – 231: The scores were interpreted to assess preparedness and implementation levels using a criteria-based rating scale based on Bloom's cut-off categories: poor (less than 60%), moderate (60 – 79%), and good (80 – 100%) [24, 25].

[24] Akalu Y, Ayelign B, Molla MD. Knowledge, Attitude and Practice Towards COVID-19 Among Chronic Disease Patients at Addis Zemen Hospital, Northwest Ethiopia. Infect Drug Resist. 2020;13: 1949–1960. https://doi.org/10.2147/IDR.S258736.

[25] Jeenmuang K, Kaewsawas S, Thanapop C, Thanapop S. Social Support, Active Ageing Perception and Practices among Educational Staff in the Primary Educational Service, Nakhon-Si-Thammarat Province, Southern Thailand. Soc Sci. 2023;12(9): 486. https://doi.org/10.3390/socsci12090486.

Comment 3.5: *Clarify if any statistical software was used for analysis.*

Response: This study used descriptive statistics to analyze the data and describe its characteristics, including mean, standard deviation, and percentage. Therefore, only a spreadsheet with essential computer software (Microsoft Excel) was utilized. No statistical software was used for analysis, so we did not mention any software in the manuscript.

4. Results

- ***Comment 4.1***: *Explain the practical significance of some percentages.*

Response: The practical significance of some percentages was explained point by point and added to the Discussion, as indicated in the lines presented in the manuscript.

1. Most of the CIFs (80.7%) in Nakhon Si Thammarat Province were established by local government organizations (LGOs), as shown in Table 1.

Line 369 – 371: "The approach of aligning responsibility to LGOs for establishing CIFs (see Fig 1) solved the problem of bed shortages for treating hospital patients."

2. Approximately one–half of the CIFs (45.6%) were classified as small CIFs (1 – 38 beds), as shown in Table 1.

Line 436 – 439 (already presented in the Discussion of the manuscript): The sizes or numbers of beds in each CIF might vary depending on factors such as the number of patients in each community willing to be admitted, the capacity of the infrastructure, and available resources, including financial support from LGOs, donations, and supporting staff.

Line 439 – 441 (added in the Discussion of revised the manuscript): Most CIFs in Thailand, except those in the Bangkok Metropolitan Region, operated under decentralized governance through LGOs. As a result, small CIFs predominated in this study.

3. Table 1: The majority (93.0%) of the CIFs in this study did not use air conditioning and relied on natural ventilation, as shown in Table 1.

Line 422 – 425 (rearrange the sentences already presented in the Discussion of the manuscript): Schools and government buildings were the pre-existing public infrastructures repurposed to establish CIFs in this study. Most were open-air structures capable of diluting infectious aerosols through ventilation with outside air, thus reducing pathogens accumulation in cases where mechanical ventilation was unavailable.

Line 425 – 427 (added in the Discussion of revised the manuscript): Open-air buildings are usually present in rural communities because of low investment and electricity costs. The installation of additional equipment would delay the immediate operation of the CIFs during an outbreak.

4. Only one–third of the CIFs (33.3%) had a system for disinfecting wastewater before discharging, as shown in Table 3.

Line 545 – 546 (added): "The effluent should be disinfected by adding chlorine before discharging into the environment."

Line 546 – 548 (already presented in the Discussion of the manuscript): "Besides the availability of infrastructures, limitations in the knowledge and experience of the staff to operate the system were suggested (see Table 2)."

Line 553 – 561 (added, as shown in the underlined phrase): "Therefore, the discharge of wastewater without appropriate treatment might increase the probability of the disease transmission. Training on wastewater disinfection for the operating staff is needed to prevent the spread of the virus to the environment."

5. All the CIFs in this study were well-prepared in terms of supplies (100%), as shown in Table 4.

Line 507 – 513: "All the CIFs in this study were well-prepared regarding supplies at a good level (see Table 4). LGOs maintain a reserve or emergency budget that can be utilized if an area is declared a disaster zone under the authority of the local government council by the decentralized structure of the Thai administrative system. This approach enables each CIF to secure sufficient funding to allocate various supplies required for operations. Furthermore, donations of essential goods, food, and medical supplies are provided to each local CIF by the local population and private organizations."

- ***Comment 4.2***: *Include p-values or statistical significance tests where relevant.*

Response: Inferential statistics was not employed in this study; therefore, no p-values or statistical significance were present.

- ***Comment 4.3***: *Analyze why there are differences in educational backgrounds.*

  Response: Thank you for this suggestion. The differences in educational backgrounds were analyzed and are presented in the Discussion section as follows.

  Line 482 – 489: The different patterns in the educational background between the founding and operating staff might be related to the organization to which they belong. Most founding staff (89.5%) were officers from LGOs, while more than half of the operating staff (50.9%) came from public health agencies. The findings of this study reflect the shortage of qualified personnel suitable to be founding staff of CIFs, which may result from some LGOs lacking departments directly involved in environmental health work. The cooperation from the Public Health Office in supporting staff participation as operating staff in CIFs was part of the effort to maintain appropriate practices in CIFs.

  Line 498 – 505: Moreover, the result also showed that most of the Master's degrees in both groups of participants were in humanities and social science fields, which might be because the postgraduate degree was beneficial for their promotion to executive positions. The founding staff, mostly from LGOs, showed a higher proportion of Master's degree holders (47.4%) than the operating staff (21.1%). The founding staff was responsible for checking the readiness of the infrastructure and personnel and setting up the systems. The postgraduate degree would benefit the management of resources and the decision-making of the founding staff. However, as already mentioned, the result showed that most of the founding staff lacked relevant knowledge and experience in environmental health.

5. Discussion

- ***Comment 5.1****: Elaborate on Thailand's unique approach to COVID-19 management.*

  Response: Thank you for this suggestion. We have added the following sentences in the Discussion section to provide more detail on Thailand's unique approach to COVID-19 management.

Line 366 – 367: "Thailand has a unique approach to managing COVID-19 using a decentralized governance model."

Line 410 – 418: "Strong underlying health systems, especially in primary healthcare, are the drivers to identify and contain COVID-19 infections and outbreaks. Village health volunteers, ordinary people who devoted themselves to helping those in their village, were recognized as an essential component of the national COVID-19 response. In CIFs, these volunteers were key in screening and inspecting infected individuals in their communities, informing community leaders, and referring patients for timely treatment or appropriate containment by HI or CI when necessary. Promptly responding to asymptomatic and mildly symptomatic COVID-19-confirmed patients is crucial for effective surveillance, early detection, and health monitoring of COVID-19 patients in Thailand."

- ***Comment 5.2****: Provide more context about the decentralized governance model.*

  Response: Thank you for this suggestion. We have added the following sentences to the Discussion section to provide more details on the decentralized governance model.

  Line 367 – 369: "The decentralized governance model allows LGOs to make decisions, manage resources and services, and respond to their area's needs. Therefore, this model could facilitate rapidly managing various crises, including the COVID-19 pandemic."

- ***Comment 5.3****: More exploration of why certain environmental health practices were suboptimal.*

  Response: Poor wastewater management is the key issue that has resulted in a moderate level of environmental health management in CIFs. The leading cause of this issue is the infrastructure of CIFs, most of which are government buildings temporarily used as COVID-19 patient care facilities. As a result, there were some CIFs (28.1%) where the wastewater treatment system was either unavailable or malfunctioning. Additionally, most staff overseeing the CIFs lacked experience in

environmental health (see Table 2) to operate the wastewater treatment system and disinfection. These issues were mentioned in the discussion section as follows.

Line 544 – 548: "This finding was consistent with the lack of wastewater treatment and disinfection systems in the infrastructure of CIFs. The effluent should be disinfected by adding chlorine before discharging into the environment. Besides the availability of infrastructures, limitations in the knowledge and experience of the staff to operate the system were suggested (see Table 2)."

In addition to wastewater management, some CIFs (15.8%) operated moderately in food sanitation. The discussion section mentions this issue as follows.

Line 562 – 573: "In addition to medical care, nutrition and hygiene are important to help the patient's recovery. Establishing a list of certified food suppliers and protocols for inspecting food from unknown sources is crucial. In some cases, nearby restaurants with short transportation times were selected. Patients should avoid food cooked with coconut milk, high-fat content, and raw ingredients. The data in Table 5 showed that most of the CIFs in this study sometimes served that kind of food to the patients, for example, coconut milk curry and salad. Coconut milk is easily spoiled within a few hours, especially in warm temperatures. As a result, it is recommended to consume food with coconut milk within a few hours. In addition, high-fat content and raw ingredients also pose health risks for patients with underlying diseases, such as cardiovascular diseases and microbial food poisoning, respectively. This study showed that these issues were particularly concerning for food from outsources or supplied by donation since delivery time could not be strictly controlled and certain types of food could not be prohibited."

- ***Comment 5.4****: Discuss potential systemic reasons for implementation challenges.*

  Response: A systemic reason that challenges the implementation is that most LGOs' organizational structures still lack a dedicated environmental health division or staff with experience in

environmental health. According to this comment, we have added this issue to the Discussion section as follows.

Line 489 – 491: "However, operations requiring assistance and consultation from the Public Health Office might cause delays in responding to urgent situations."

- ***Comment 5.5***: *Expand on comparisons with CIF strategies in other countries.*

  Response: Thank you for this suggestion. We have added the following sentences to the Discussion section to emphasize the uniqueness of the CIF strategy in Thailand.

  Line 371 – 409: "Compared to other countries, they employed centralized strategies, and national health authorities played a significant role in the decision-making and allocating of resources, which differed from Thailand. This study showed that Thailand's decentralized governance model facilitated small CIFs' operation in local communities, with LGOs playing a significant role (see Table 1)."

- ***Comment 5.6***: *Expand on study limitations.*

  Response: Thank you for this suggestion. We have added the following sentences in the Discussion section to state the study limitation.

  Line 636 – 643: "This research could not be conducted during the outbreak period, when the staff were working, due to restrictions on access to CIFs. Additionally, the staff had to be quarantined because of their close contact with infected individuals and could not meet others. As a result, the researchers could not collect data on the environment within the CIFs during the operation and had to rely on retrospective data from the staff after the outbreak. Therefore, providing suggestions or feedback for improvements during the operational period was impossible. Furthermore, there was a lack of specific data on the number of infected individuals in each community, making it difficult to assess the effectiveness of CIFs' operations."

- ***Comment 5.7***: *Provide more forward-looking.*

  Response: Agree. We have added the discussion according to this comment.

  Line 694 – 696: "The knowledge of environmental health among LGO staff is valuable not only during a crisis but also for local agencies responsible for public welfare, as environmental health is an important health determinant."

Comments from Reviewer 2

General Comments: It is a valuable study, but some corrections are needed.

- ***Comment 1***: *Items related to sampling and people's characteristics should be discussed in the method.*

  Response: Items related to sampling have already been added to the method as follows.

  Line 148 – 150: "During the study period, there were 114 CIFs in the province. Out of these, 57 CIFs met the criteria and consented to participate in the study. Accordingly, 57 founding and 57 operating staff members were enrolled in the study."

  Response: For the people's characteristics, we think that the results should address the people's characteristics as they were obtained from the questionnaires.

- ***Comment 2****:* The discussion is long, and some content should be included in the introduction, methodology and results.

  Response: The following content has been moved to the Introduction.

  Line 97 – 104: "Thailand is the only upper-middle-income country in the top five of the Global Health Security (GHS) Index among 195 countries in 2021. This high ranking reflects the effectiveness of the government's public health policy and emergency management during the COVID-19 pandemic.

Prompt response to patients with mild or asymptomatic symptoms of COVID-19 by adopting the CI strategy played a significant role in effective disease control in Thailand. The containment of COVID-19 patients in CIFs could suppress the spread of SARS-CoV-2 to the community by preventing transmission via direct contact with the patients and indirect contact through environmental pathways."

Response: The following content and Fig 1 have been moved to the Methodology.

Line 125 – 142: "The operation of CIFs in Thailand was initiated by the Centre for COVID-19 Situation Administration (CCSA) under the Emergency Decree on Public Administration in Emergency Situations, B.E. 2548. The Prime Minister directly commanded the CCSA, working with the Ministry of Public Health and related government agencies. The levels of organizations involved in establishing and operating the CIFs are demonstrated in Fig 1. The establishment of the CIFs in each province was managed under orders from the Permanent Secretary of the Ministry of Interior. The Provincial Governor put measures in place for the LGOs to act as the host of CIFs. In addition, local public health offices also acted as the host and cooperated with LGOs to operate the CIFs."

We look forward to hearing from you in due time regarding our submission and to respond to any further questions and comments you may have.

Sincerely,

Udomratana Vattanasit

Udomratana Vattanasit
